# NAT10 promotes radiotherapy resistance in non-small cell lung cancer by regulating KPNB1-mediated PD-L1 nuclear translocation

**DOI:** 10.1515/biol-2025-1065

**Published:** 2025-03-18

**Authors:** Dagao Zhu, Mingliang Lu, Hongmin Cheng

**Affiliations:** Department of Radiation Oncology, The Affiliated Tongling Hospital of Bengbu Medical University, No. 468 Bijiashan Road, Tongguan District, Tongling, 244000, China; Department of Radiation Oncology, The People’s Hospital of Tongling City, No. 468 Bijiashan Road, Tongguan District, Tongling, 244000, China

**Keywords:** non-small cell lung cancer, radiotherapy resistance, immune escape, NAT10, ac4C, KPNB1

## Abstract

Radiotherapy (RT) resistance in non-small cell lung cancer (NSCLC) is a significant contributor to tumor recurrence. NAT10, an enzyme that catalyzes ac4C RNA modification, has an unclear role in RT resistance. This study aimed to explore the function of NAT10 in RT resistance in NSCLC. RT-resistant NSCLC cell lines (PC9R and A549R) were established through repeated irradiation. The impact of NAT10 on cellular immunity was evaluated by measuring immune cell populations, cytotoxicity levels, and markers of cell dysfunction. Results demonstrated elevated levels of ac4C and NAT10 in RT-resistant cells. Knockdown of NAT10 suppressed cell proliferation and enhanced immune function in PC9R and A549R cells by upregulating TNF-α and IFN-γ while downregulating PD-1 and TIM-3. Mechanistically, RT resistance in NSCLC was mediated by NAT10-dependent ac4C modification of KPNB1. Furthermore, KPNB1 facilitated PD-L1 nuclear translocation, promoting immune escape in RT-resistant NSCLC cells. Overexpression of KPNB1 enhanced cell proliferation but impaired immune function in RT-resistant NSCLC cells. In conclusion, this study demonstrates that NAT10 upregulates KPNB1 expression through ac4C modification, thereby promoting RT resistance in NSCLC via PD-L1 nuclear translocation. These findings reveal a novel mechanism underlying RT resistance in NSCLC.

## Graphical abstract

**Figure:**
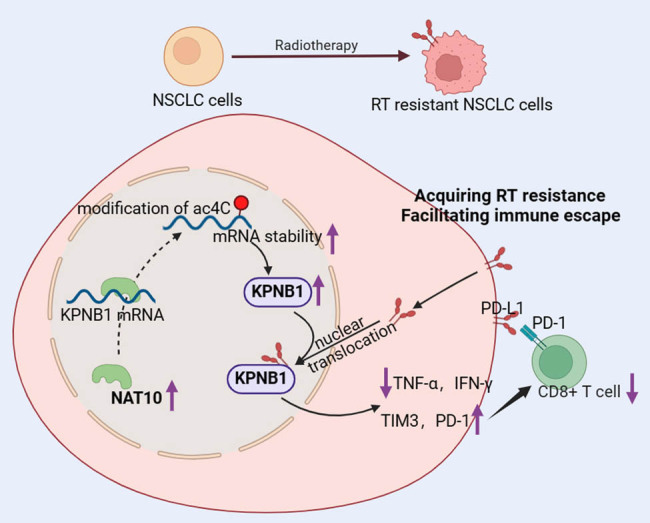

## 1 Introduction

Non-small cell lung cancer (NSCLC) constitutes approximately 80–85% of all lung cancer cases and remains one of the leading causes of cancer-related mortality worldwide. Based on histopathological characteristics, NSCLC is primarily classified into two major subtypes: lung adenocarcinoma (LUAD) and lung squamous cell carcinoma (LUSC) [1]. Current therapeutic strategies for NSCLC include surgery, chemotherapy, radiotherapy (RT), targeted therapy, immunotherapy, or a combination of these modalities, which have collectively contributed to improved survival rates among patients [2–4]. Among these, RT serves as a cornerstone treatment for NSCLC. Its mechanism of action involves inducing lethal DNA damage either directly in irradiated cells or indirectly through the generation of reactive oxygen species [5]. Notably, approximately 77% of NSCLC patients have evidence-based indications for RT, and this treatment has been demonstrated to significantly enhance clinical outcomes and overall survival rates [6,7]. Despite these advancements, a subset of patients experiences localized tumor recurrence following standard clinical doses of radiation, which may be attributed to the development of RT resistance in tumor cells [8]. This resistance poses a significant challenge to the efficacy of RT and underscores the need to elucidate its underlying mechanisms. Understanding the molecular basis of RT resistance is crucial for developing strategies to overcome this limitation, thereby improving therapeutic outcomes and prolonging survival in NSCLC patients.

RT resistance represents a major contributor to tumor treatment failure and metastasis [9]. The mechanisms underlying RT resistance are highly complex and remain incompletely understood. Current evidence suggests that RT resistance is influenced by a multitude of factors, including genetic alterations, the tumor microenvironment, and metabolic reprogramming such as aerobic glycolysis [10,11]. In NSCLC specifically, RT resistance has been linked to epigenetic modifications, dysregulation of non-coding RNAs, and imbalances in ferroptosis [12–14]. Notably, emerging research highlights the critical role of immune dysfunction in mediating RT resistance. For instance, RT resistance has been shown to impair the expansion of tumor-antigen-specific T cells and suppress the activation of immune priming mechanisms [15]. Additionally, RT resistance promotes the upregulation of immune checkpoint molecules, such as CD47 and PD-L1, which facilitate tumor metastasis and immune evasion [16]. Furthermore, studies have demonstrated that immunosuppressive effects, including PD-L1 upregulation and enhanced glucose metabolism in neutrophils, play pivotal roles in driving RT resistance in NSCLC [17,18]. Despite these advances, the mechanisms of RT resistance in NSCLC remain poorly characterized, and there is an urgent need to identify novel therapeutic targets to sensitize tumors to radiation and overcome resistance.

NAT10, a crucial member of the Gcn5-related N-acetyltransferase family, is the only known “writer” protein responsible for ac4C RNA modification, catalyzing the addition of ac4C to mRNA [19]. This NAT10-mediated ac4C modification plays a pivotal role in enhancing mRNA stability and increasing translation efficiency. Through its acetyltransferase activity, NAT10 regulates various cellular processes, including apoptosis, autophagy, and cell proliferation [20]. Accumulating evidence has highlighted the significance of NAT10 in cancer biology, where it promotes tumor initiation, metastasis, and progression by catalyzing ac4C modifications on mRNA, thereby enhancing translational efficiency and driving the expression of oncogenic genes [21–23]. In NSCLC specifically, NAT10 has been shown to be upregulated and is associated with poor patient prognosis [24,25]. Despite these findings, the role of NAT10 in mediating RT resistance in NSCLC remains poorly understood and warrants further investigation.

Despite these findings, the role of NAT10 in mediating RT resistance in NSCLC remains poorly understood and warrants further investigation. Elucidating the mechanisms by which NAT10 contributes to RT resistance could provide critical insights into overcoming therapeutic challenges and improving outcomes for NSCLC patients.

## 2 Methods

### 2.1 Cell culture and irradiation treatment

The LUAD cell line PC9 and the human NSCLC cell line A549 were obtained from the American Type Culture Collection (Manassas, VA, USA). Cells were cultured in RPMI-1640 medium (Gibco, Grand Island, NY, USA) supplemented with 10% FBS (Gibco) and 1% penicillin/streptomycin (Gibco) at 37°C in a humidified incubator with 5% CO₂. Radiation-resistant cell lines were generated following a previously described method [26]. Briefly, PC9 and A549 cells were seeded into 60 mm dishes and irradiated using a ^137^Cs source when they reached approximately 60% confluency. Cells were exposed to 2 Gy of radiation once daily until a cumulative dose of 100 Gy was achieved. The resulting radiation-resistant cell lines were designated as PC9R and A549R. To assess radiation resistance, a subset of PC9R and A549R cells was further irradiated with 6 Gy and incubated for 72 h. This dose was administrated to parental NSCLC cells, and results confirmed that 6 Gy irradiation significantly inhibited cell viability and proliferation while promoting apoptosis in parental PC9 and A549 cells (Figure S1a–e).

### 2.2 Cell transfection

Short hairpin RNAs targeting NAT10 (shNAT10) and shKPNB1, along with a negative control shRNA (shNC), were purchased from GenePharma (Shanghai, China). Additionally, KPNB1 overexpression plasmids (pcDNA3.1-KPNB1) and the corresponding empty vector control (pcDNA3.1-vector) were obtained from the same supplier. Transfection was performed using Lipofectamine 2000 (Invitrogen, Carlsbad, CA, USA) according to the manufacturer’s protocol. Briefly, A549R and PC9R cells in the logarithmic growth phase were transfected, and cells were harvested 48 h post-transfection for subsequent analysis.

### 2.3 Cell viability assay

Cell viability was assessed using a Cell Counting Kit-8 (CCK-8; Yeasen, Shanghai, China) according to the manufacturer’s instructions. Briefly, cells were seeded into 96-well plates at an appropriate density and cultured under standard conditions. After the designated treatment, 10 µL of CCK-8 solution was added to each well, and the plates were incubated at 37°C for 2 h. The absorbance of each well was measured at 450 nm using a microplate reader (BioTek, Winooski, VT, USA).

### 2.4 Colony formation assay

The colony formation assay was performed to assess cell proliferation capacity. Cells were seeded into six-well plates at a density of 500 cells per well and cultured at 37°C in a humidified incubator with 5% CO₂. After 14 days of incubation, cells were fixed with 4% paraformaldehyde for 15 min and then stained with 0.2% crystal violet solution for 30 min at room temperature. Colonies containing more than 50 cells were counted manually under a microscope.

### 2.5 Detection of apoptosis

Apoptosis in PC9R and A549R cells was assessed using an Annexin V-FITC/PI Apoptosis Detection Kit (Beyotime, Shanghai, China) according to the manufacturer’s instructions. Briefly, cells were harvested, washed twice with PBS, and centrifuged at 1,000*g* for 5 min to remove the supernatant. The cell pellet was resuspended in Annexin V-FITC binding buffer and stained with Annexin V-FITC and PI for 20 min at room temperature in the dark. Apoptotic cells were analyzed using a flow cytometer (BD Biosciences, San Jose, CA, USA). The percentages of early apoptotic (Annexin V-FITC-positive/PI-negative) and late apoptotic (Annexin V-FITC-positive/PI-positive) cells were quantified.

### 2.6 Dot blot assay

The levels of ac4C modification in PC9 and A549 cells were measured using a dot blot assay. Total RNA was extracted from PC9 and A549 cells using Trizol reagent (Invitrogen, Carlsbad, CA, USA) according to the manufacturer’s instructions. The isolated RNA was denatured by heating at 65°C for 5 min and then spotted onto a Hybond-N + membrane (GE Healthcare, Chicago, IL, USA). The RNA was immobilized on the membrane by UV cross-linking for 5 min. Subsequently, the membrane was blocked with 5% skim milk in Tris-buffered saline with Tween-20 for 1 h at room temperature to prevent nonspecific binding. The membrane was then incubated with a primary anti-ac4C antibody overnight at 4°C, followed by incubation with a horseradish peroxidase (HRP)-conjugated secondary antibody for 1 h at room temperature. Finally, the signal was detected using an enhanced chemiluminescence (ECL) kit (Thermo Scientific, Waltham, MA, USA) and visualized using a chemiluminescence imaging system.

### 2.7 Quantitative real-time PCR (qPCR)

RNA isolation was conducted using Trizol reagent and reverse transcribed into cDNA using a HiScript II first strand cDNA synthesis kit (Vazyme, Nanjing, China). qPCR was performed using SYBR green reagent and the mixture was prepared followed by the manufacturer’s protocol. The relative mRNA expression was calculated using the 2^−∆∆Ct^ method with GAPDH serving as the internal reference. The qPCR primers used in this study were as follows: NAT10, 5′-ATAGCAGCCACAAACATTCGC-3′(forward) and 5′-ACACACATGCCGAAGGTATTG-3′ (reverse); TNF-α, 5′-CCTCTCTCTAATCAGCCCTCTG-3′(forward) and 5′-GAGGACCTGGGAGTAGATGAG-3′(reverse); IFN-γ, 5′-TCGGTAACTGACTTGAATGTCCA-3′(forward) and 5′-TCGCTTCCCTGTTTTAGCTGC-3′(reverse); PD-1, 5′-CCAGGATGGTTCTTAGACTCCC-3′(forward) and 5′-TTTAGCACGAAGCTCTCCGAT-3′(reverse); TIM3, 5′-CTGCTGCTACTACTTACAAGGTC-3′(forward) and 5′-GCAGGGCAGATAGGCATTCT-3′(reverse); KPNB1, 5′-CCACTTTCCTTGTGGAACTGT-3′(forward) and 5′-CTCTGCTGATATTGTGCCTTGA-3′ (reverse); NCL, 5′-GGTGGTCGTTTCCCCAACAAA-3′(forward) and 5′-GCCAGGTGTGGTAACTGCT-3′ (reverse).

### 2.8 Immunofluorescence (IF) staining

IF staining was performed using an IF staining kit (Beyotime, Shanghai, China) according to the manufacturer’s protocol. Briefly, cells were fixed with 4% paraformaldehyde (included in the kit) overnight at 4°C. After fixation, cells were washed three times with PBS containing 0.1% Tween-20 (PBST) to remove residual fixative. Cells were then blocked with blocking buffer (provided in the kit) for 1 h at room temperature to prevent nonspecific binding. Subsequently, cells were incubated overnight at 4°C with primary antibodies against NAT10, KPNB1, or PD-L1. After washing with PBST, cells were incubated with fluorescence-conjugated secondary antibodies for 1 h at room temperature in the dark. Finally, cells were counterstained with 4′,6-diamidino-2-phenylindole to visualize nuclei and imaged using a fluorescence microscope (Nikon, Tokyo, Japan).

### 2.9 Co-culture of T cells and tumor cells

Peripheral blood mononuclear cells (PBMCs) were obtained from BLUEFBIO (Shanghai, China). T cells were isolated using the Dynabeads^®^ Untouched™ Human T Cells Kit (Thermo Scientific, Waltham, MA, USA) according to the manufacturer’s instructions. The co-culture of T cells and tumor cells was performed as previously described [27]. Briefly, PBMCs were seeded into six-well plates at a density of 2 × 10^6^ cells per well and stimulated with cell lysates derived from PC9R or A549R cells, along with anti-CD3ε and anti-CD28 antibodies, to activate T cells. Activated T cells were then co-cultured with PC9R or A549R cells at a 10:1 ratio (T cells:tumor cells) for 16 h. Cells were incubated with PE-conjugated anti-CD4 and APC-conjugated anti-CD8 antibodies for 30 min at 4°C. The proportions of CD4^+^ and CD8^+^ T cells were analyzed by flow cytometry. Additionally, the expression levels of TNF-α, IFN-γ, PD-1, and TIM-3 in the co-culture system were quantified using qPCR.

### 2.10 Bioinformatic analysis

Genes associated with NAT10 in LUAD and LUSC were screened using the LinkedOmics database (https://www.linkedomics.org/). Genes meeting the criteria of *p* < 0.05 and |*r*| > 0.4 (*r* > 0.4 or *r* < −0.4) in both LUAD and LUSC were selected and intersected. Pathway enrichment analysis was subsequently performed on these overlapping genes using FunRich software. Additionally, the correlations between KPNB1 and NAT10, as well as between NCL and NAT10, were analyzed using the LinkedOmics database.

### 2.11 Methylated RNA immunoprecipitation (MeRIP)

The ac4C modification level on KPNB1 in PC9R and A549R cells was assessed using a GenSeq ac4C RIP kit (Cloudseq, Shanghai, China) according to the manufacturer’s instructions. Briefly, RNA was fragmented using a fragmentation buffer on a PCR instrument, followed by incubation at 70°C for 6 min. Protein G magnetic (PGM) beads were pre-incubated with anti-ac4C antibody for 1 h at room temperature. The fragmented RNA was then incubated with the antibody-coated PGM beads for 1 h on a rotator at 4°C. After incubation, the beads were washed and purified following the manufacturer’s protocol. The ac4C modification level of KPNB1 was subsequently quantified by qPCR.

### 2.12 RIP

The interaction between NAT10 and KPNB1 was investigated using an RIP assay kit (Beyotime, China). Briefly, protein A/G agarose beads were incubated with anti-KPNB1 antibody or anti-IgG (negative control) for 30 min at room temperature. Cells were lysed using a specific lysis buffer for 15 min on ice, followed by centrifugation at 14,000*g* for 10 min at 4°C to collect the supernatant. The antibody-coated protein A/G agarose beads were then incubated with the supernatant for 4 h at 4°C on a shaker. After incubation, the samples were eluted and purified according to the manufacturer’s protocol. The expression of KPNB1 was subsequently quantified by qPCR.

### 2.13 RNA stability assay

To assess the stability of KPNB1 mRNA in PC9R and A549R cells, cells were treated with 5 μg/mL actinomycin D (Merck, Darmstadt, Germany) to inhibit transcription. The expression levels of KPNB1 were measured by qPCR at baseline (0 h) and at 4, 8, and 12 h post-treatment.

### 2.14 Dual luciferase reports

Wild-type (wt) and mutant (mut) sequences containing potential KPNB1 binding sites were cloned into the pGL3 vector to construct luciferase reporter plasmids. Transfection was performed in PC9R and A549R cells when they reached approximately 80% confluency, using Lipofectamine 2000 reagent (Invitrogen, Carlsbad, CA, USA) according to the manufacturer’s instructions. After 48 h of transfection, luciferase activity was measured using a dual-luciferase reporter assay system (Promega, San Luis Obispo, CA, USA).

### 2.15 Co-immunoprecipitation (co-IP)

The interaction between KPNB1 and PD-L1 was examined using a Pierce Classic Magnetic IP/Co-IP kit (Thermo Scientific, USA). Cells were lysed with IP lysis buffer on ice for 5 min, followed by centrifugation to collect the supernatant. The lysate was incubated overnight at 4°C with anti-KPNB1, anti-PD-L1, or anti-IgG (negative control) antibodies. Subsequently, the samples were incubated with protein A/G magnetic beads for 1 h at room temperature. After washing, the immunoprecipitated complexes were eluted according to the manufacturer’s protocol. The protein levels of KPNB1 and PD-L1 were analyzed by western blot.

### 2.16 Western blot

Total protein was extracted from PC9R and A549R cells using RIPA lysis buffer (Beyotime, China). Nuclear protein isolation was performed using a nuclear protein extraction kit (Beyotime, China) according to the manufacturer’s instructions. Protein samples were separated on 10% SDS-PAGE gels and transferred to PVDF membranes. The membranes were blocked with 5% skimmed milk for 1 h at room temperature and then incubated with primary antibodies overnight at 4°C. The primary antibodies used in this study included anti-KPNB1 (1:1,000, ab313370; Abcam, Cambridge, MA, USA), anti-PD-L1 (1:1,000, ab213524; Abcam), anti-GAPDH (1:10,000, ab181602; Abcam), and anti-Lamin B1 (1:10,000, ab133741; Abcam). After incubation, the membranes were washed and incubated with HRP-conjugated secondary antibodies (1:10,000, ab6721; Abcam) for 2 h at room temperature. Protein bands were visualized using an ECL reagent.

### 2.17 Statistical analysis

All experiments were independently repeated at least three times, and the data are presented as mean ± SD. Statistical analyses were performed using GraphPad Prism 7.0 software (GraphPad Software, San Diego, CA, USA). Differences between two groups were assessed using an unpaired Student’s *t*-test, while comparisons among multiple groups were analyzed by one-way analysis of variance. A *p*-value of less than 0.05 (*p* < 0.05) was considered statistically significant.

## 3 Results

### 3.1 RT leads to therapeutic resistance in NSCLC cells

**Figure 1:**
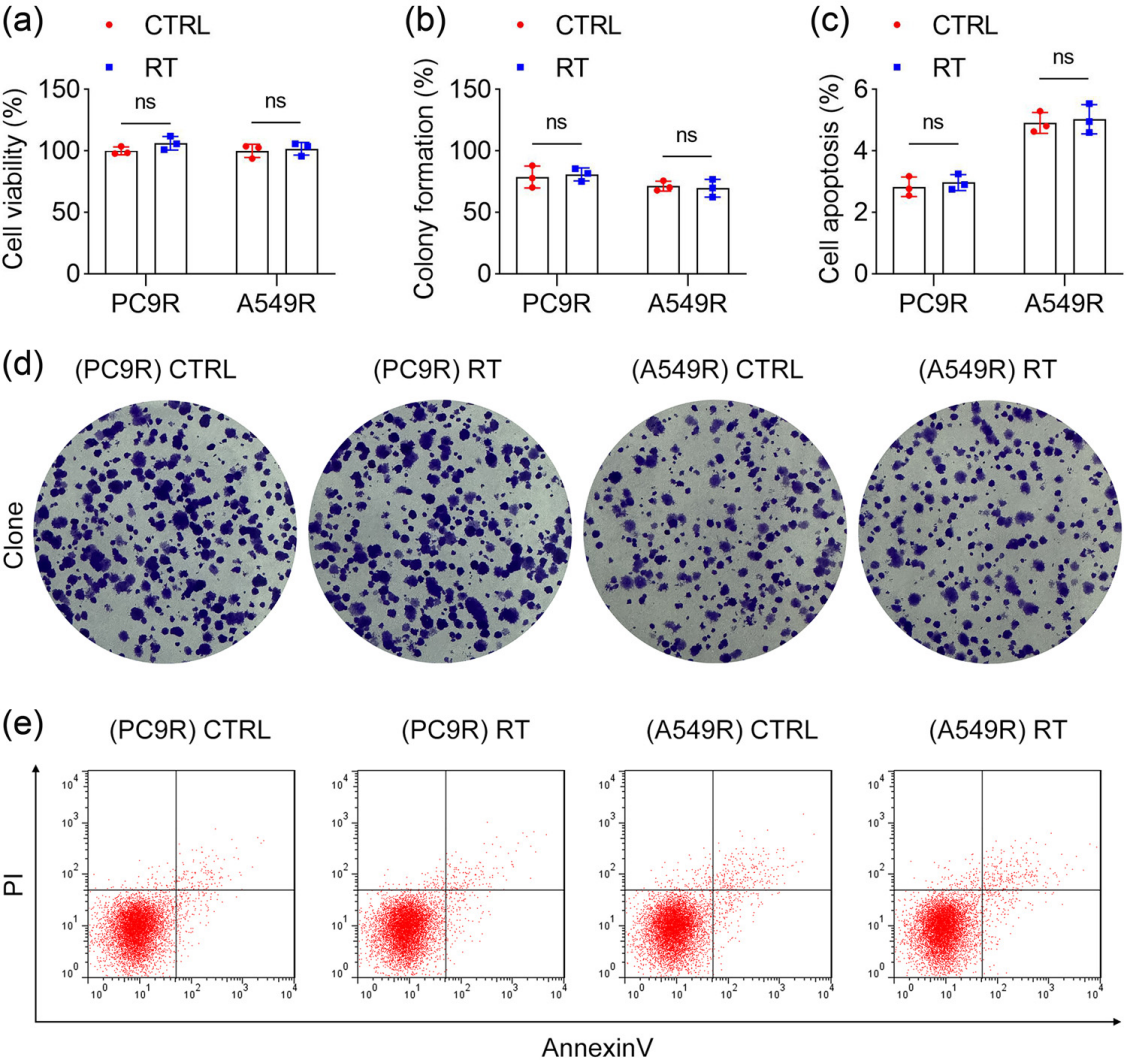
RT led to therapeutic resistance in NSCLC cells. (a) Cell viability of PC9R and A549R cells was detected using a CCK8 kit. (b) and (d) Cell proliferation ability of PC9R and A549R cells was evaluated by colony formation assay. (c) and (e) Cell apoptosis of PC9R and A549R cells was detected by flow cytometry.

To determine whether RT contributes to therapeutic resistance in NSCLC cells, radiation-resistant PC9R and A549R cells were subjected to additional irradiation. Therapeutic resistance was assessed by evaluating cell viability, proliferation, and apoptosis. The results demonstrated no statistically significant differences in cell viability (Figure 1a), proliferation (Figure 1b and d), or apoptosis (Figure 1c and e) between RT-treated and untreated PC9R and A549R cells. These findings suggest that RT induces therapeutic resistance in NSCLC cells.

### 3.2 ac4C modification and NAT10 expression are enhanced in radiation-resistant NSCLC cells

**Figure 2:**
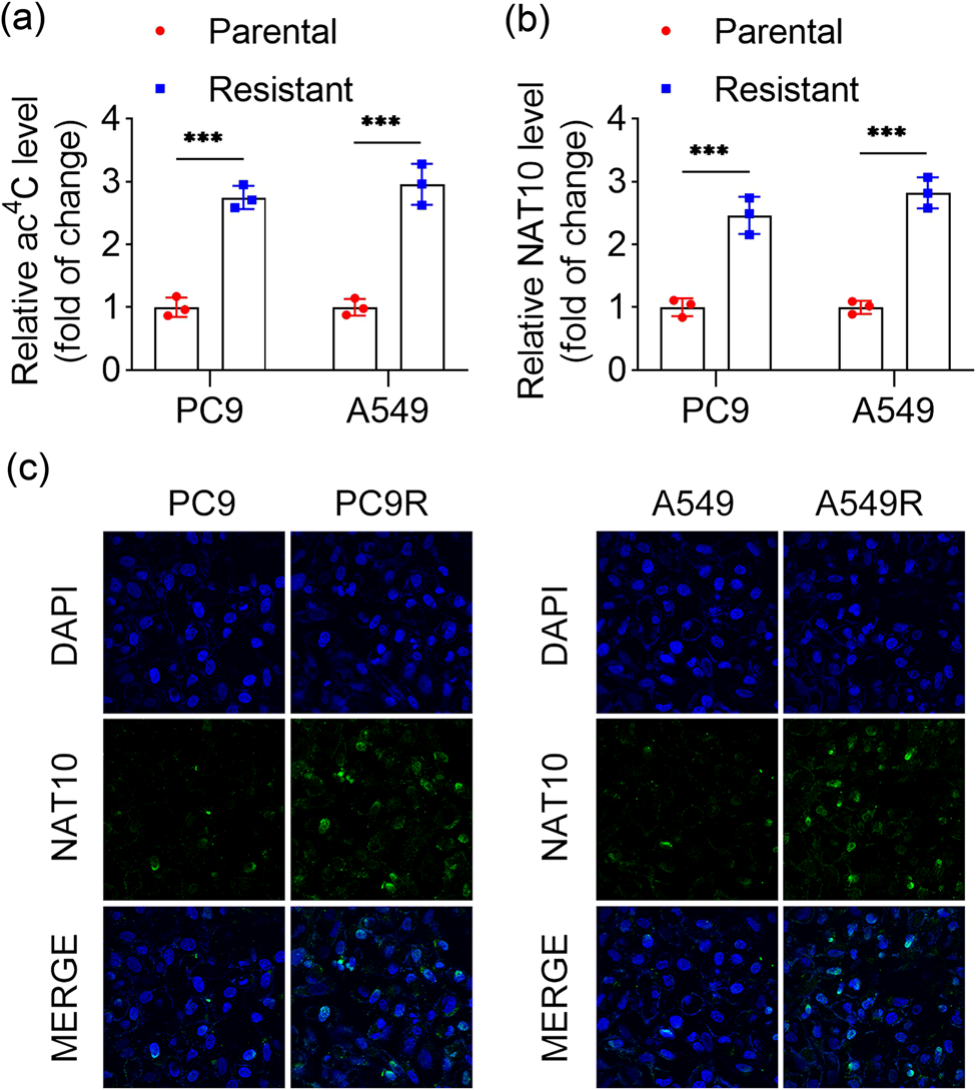
ac4C modification and NAT10 expression were enhanced in radiation-resistant NSCLC cells. (a) ac4C levels in PC9 and A549 cells were detected by dot blot assay. (b) NAT10 expression in PC9 and A549 cells was measured by qPCR. (c) IF staining showed the expression of NAT10 in PC9R and A549R cells.

To explore the role of ac4C modification in RT resistance in NSCLC, we compared the levels of ac4C modification and NAT10 expression between parental (PC9 and A549) and radiation-resistant (PC9R and A549R) cells. The results revealed a significant increase in both ac4C modification levels and NAT10 expression in PC9R and A549R cells compared to their parental counterparts (Figure 2a and b). Furthermore, IF staining demonstrated enhanced fluorescence intensity of NAT10 in PC9R and A549R cells (Figure 2c). These findings indicate that ac4C modification and NAT10 expression are upregulated in radiation-resistant NSCLC cells.

### 3.3 NAT10 knockdown improves RT resistance in radiation-resistant NSCLC cells

**Figure 3:**
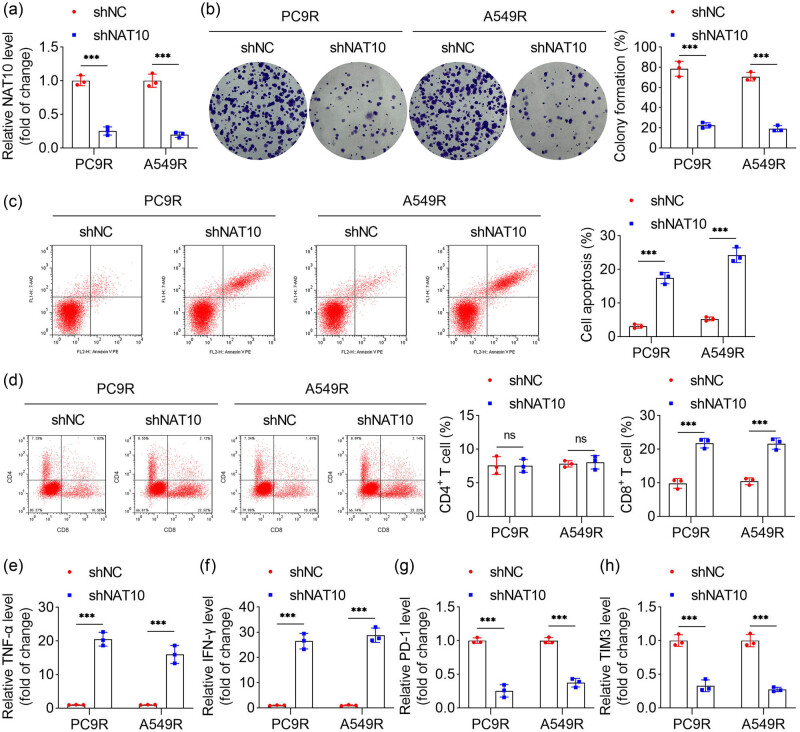
NAT10 knockdown improved RT resistance in radiation-resistant NSCLC cells. (a) qPCR was performed to measure the expression of NAT10 in PC9R and A549R cells. (b) Cell proliferation ability of PC9R and A549R cells was evaluated by colony formation assay. (c) Apoptosis of PC9R and A549R cells was evaluated by flow cytometry. (d) The amount of CD4^+^ and CD8^+^ T cells was detected by flow cytometry. (e)–(h) The levels of TNF-α, IFN-γ, PD-1, and TIM3 were measured by qPCR.

To investigate the functional role of NAT10 in RT resistance in NSCLC cells, rescue experiments were conducted. NAT10 expression in radiation-resistant NSCLC cells was significantly reduced following transfection with shNAT10 (Figure 3a). NAT10 knockdown markedly inhibited the proliferation of PC9R and A549R cells (Figure 3b) and promoted apoptosis in these cells (Figure 3c). Flow cytometry analysis revealed that NAT10 knockdown significantly increased the proportion of CD8^+^ T cells but had no effect on CD4^+^ T cell populations, suggesting that the attenuation of RT resistance by NAT10 knockdown is mediated through CD8^+^ T cells (Figure 3c). Furthermore, NAT10 knockdown elevated the levels of TNF-α and IFN-γ (Figure 3d and e) while reducing the expression of PD-1 and TIM-3 in the co-culture system (Figure 3f and g). Similar effects were observed in parental PC9 and A549 cells, where NAT10 knockdown increased CD8^+^ T cell populations and levels of TNF-α and IFN-γ but decreased PD-1 and TIM-3 expression (Figure S2a–c). Collectively, these findings demonstrate that NAT10 knockdown suppresses cell proliferation, enhances immune function, and attenuates RT resistance in radiation-resistant NSCLC cells.

### 3.4 NAT10 knockdown inhibits KPNB1 expression by decreasing ac4C modification on KPNB1 in radiation-resistant NSCLC cells

**Figure 4:**
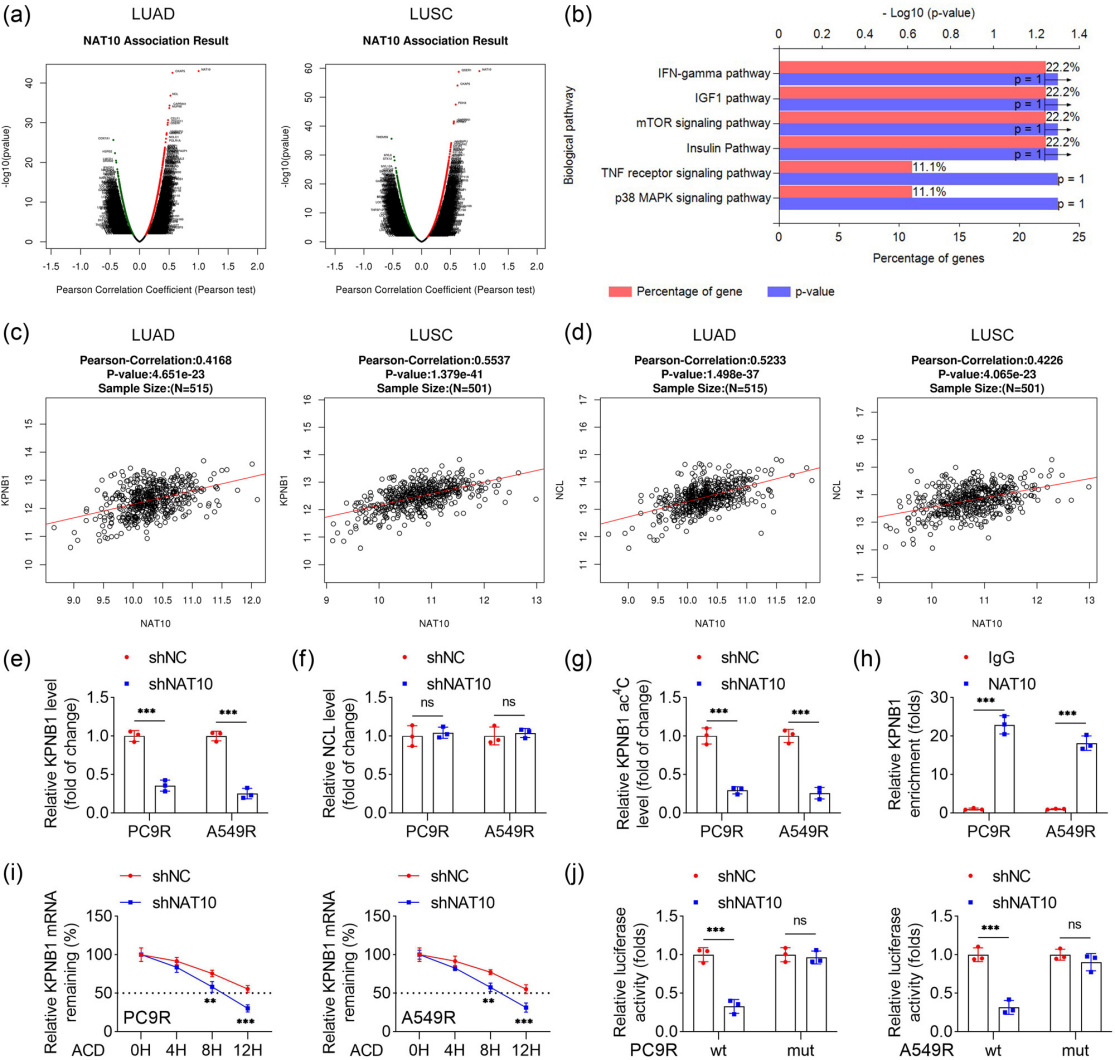
NAT10 knockdown inhibited KPNB1 expression by decreasing ac4C modification on KPNB1 in radiation-resistant NSCLC cells. (a) Genes related to NAT10 in LUAD and LUSC were screened using the LinkedOmics database. (b) Pathway enrichment analysis was conducted using Funrich software. (c) and (d) Correlation between genes related to NAT10 and NAT10 was analyzed by the LinkedOmics database. (e) and (f) Expression of KPNB1 and NCL in PC9R and A549R cells was measured by qPCR. (g) The ac4C levels on KPNB1 in PC9R and A549R cells were detected by MeRIP. (h) Interaction between NAT10 and KPNB1 was evaluated by RIP. (i) Stability of KPNB1 mRNA in PC9R and A549R cells was evaluated by qPCR before and 4, 8, and 12 h after 5 μg/mL actinomycin D treatment. (j) Luciferase activity of wt-KPNB1 and mut-KPNB1 in PC9R and A549R cells was measured by dual luciferase report.

To elucidate the mechanism by which NAT10 mediates RT resistance in NSCLC cells, we analyzed genes associated with NAT10 in LUAD and LUSC using the LinkedOmics database (Figure 4a). Pathway enrichment analysis of the intersecting genes from LUAD and LUSC revealed the IFN-γ pathway as the most significantly enriched pathway (Figure 4b). KPNB1 and NCL, both enriched in the IFN-γ pathway, showed a positive correlation with NAT10 in LUAD and LUSC (Figure 4c and d). To determine which gene interacts with NAT10 in radiation-resistant NSCLC cells, we assessed the expression of KPNB1 and NCL. NAT10 knockdown significantly reduced KPNB1 expression in PC9R and A549R cells but had no effect on NCL expression (Figure 4e and f). Furthermore, NAT10 knockdown decreased ac4C modification levels in PC9R and A549R cells (Figure 4g). RIP confirmed the interaction between NAT10 and KPNB1 (Figure 4h). NAT10 knockdown also accelerated the degradation of KPNB1 mRNA, indicating reduced stability of KPNB1 transcripts (Figure 4i). Dual-luciferase reporter assays demonstrated that NAT10 knockdown significantly reduced the luciferase activity of wt KPNB1 but had no effect on mut KPNB1 in radiation-resistant NSCLC cells (Figure 4j). Collectively, these findings demonstrate that NAT10 knockdown suppresses KPNB1 expression by reducing ac4C modification on KPNB1 mRNA in radiation-resistant NSCLC cells.

### 3.5 KPNB1 promotes radiation-resistant NSCLC cells immune escape by promotion of PD-L1 nuclear translocation

**Figure 5:**
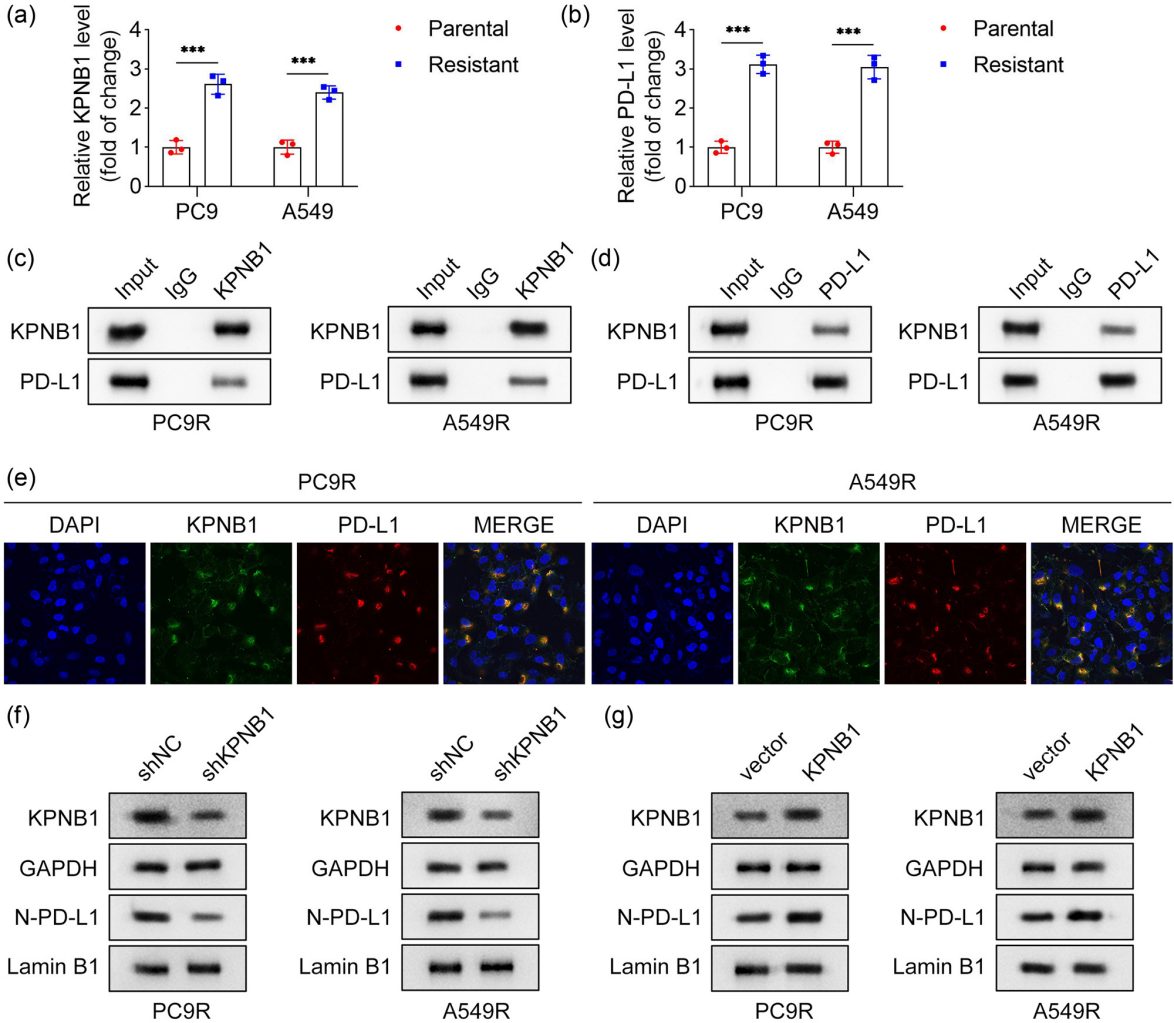
KPNB1 promoted radiation-resistant NSCLC cells immune escape by promotion of PD-L1 nuclear translocation. (a) and (b) Expression of KPNB1 and PD-L1 in parental and resistant PC9 and A549 cells was measured by qPCR. (c) and (d) The interaction between KPNB1 and PD-L1 was detected by Co-IP. (e) The location of KPNB1 and PD-L1 in PC9R and A549R cells was identified by IF staining. (f) and (g) The protein levels of KPNB1 in cells and PD-L1 in nuclear in PC9R and A549R cells were detected by western blot.

Our previous findings indicated that NAT10 knockdown downregulated nuclear PD-L1 protein levels in PC9R and A549R cells (Figure S3). Given that PD-L1 is a critical mediator of immune escape and its nuclear translocation enhances immune evasion in tumor cells, we investigated whether KPNB1, a nuclear transport factor, directly regulates PD-L1 nuclear translocation in radiation-resistant NSCLC cells. Results showed that both KPNB1 and PD-L1 expression levels were significantly higher in PC9R and A549R cells compared to their parental counterparts (Figure 5a and b). Co-IP confirmed the physical interaction between KPNB1 and PD-L1 in PC9R and A549R cells (Figure 5c and d). IF staining further demonstrated co-localization of KPNB1 and PD-L1 in these cells (Figure 5e). To assess the functional role of KPNB1 in PD-L1 nuclear translocation, we examined the effects of KPNB1 modulation. KPNB1 knockdown significantly reduced nuclear PD-L1 protein levels in PC9R and A549R cells (Figure 5d), whereas KPNB1 overexpression increased nuclear PD-L1 levels (Figure 5e), indicating that KPNB1 facilitates PD-L1 nuclear entry in radiation-resistant NSCLC cells. Furthermore, overexpression of nuclear PD-L1 in PC9R and A549R cells significantly decreased the proportion of CD8^+^ T cells, suggesting enhanced immune escape (Figure S4a–c). Together, these findings demonstrate that KPNB1 promotes immune escape in radiation-resistant NSCLC cells by facilitating PD-L1 nuclear translocation.

### 3.6 KPNB1 overexpression restores RT resistance in NSCLC cells improved by NAT10 knockdown

**Figure 6:**
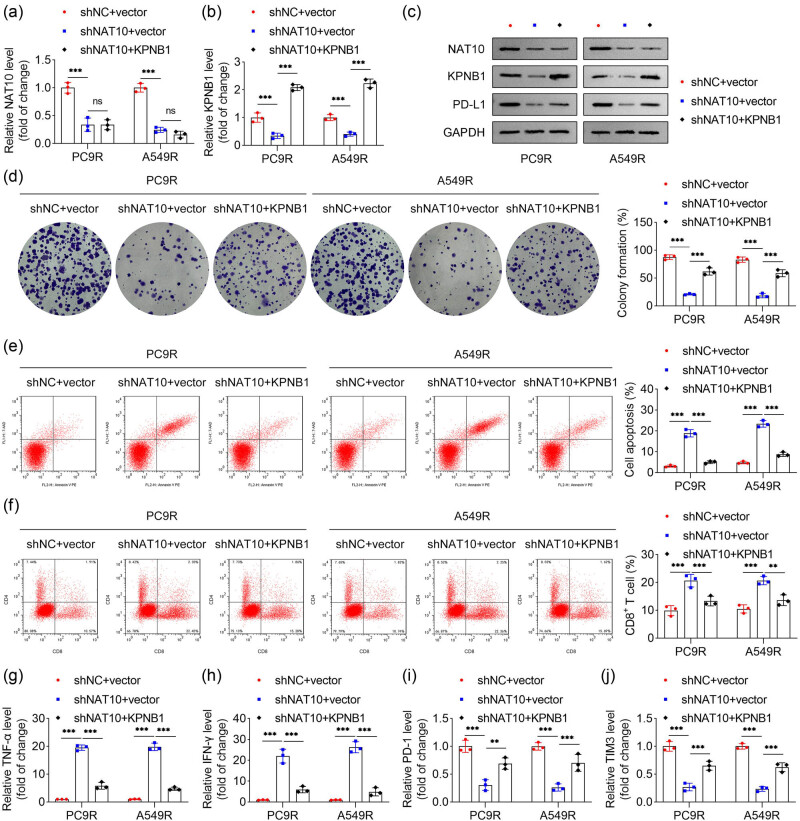
KPNB1 overexpression restored RT resistance in NSCLC cells improved by NAT10 knockdown. (a) and (b) Expression of NAT10 and KPNB1 of PC9R and A549R cells was measured by qPCR. (c) The protein levels of NAT10, KPNB1, and PD-L1 of PC9R and A549R cells were detected by western blot. (d) Cell proliferation ability of PC9R and A549R cells was evaluated by colony formation assay. (e) Apoptosis of PC9R and A549R cells was evaluated by flow cytometry. (f) The amount of CD4^+^ and CD8^+^ T cells was detected by flow cytometry. (g)–(j) The levels of TNF-α, IFN-γ, PD-1, and TIM3 were measured by qPCR.

To investigate the role of KPNB1 in RT resistance in NSCLC cells, we examined the effects of NAT10 knockdown and KPNB1 overexpression. NAT10 knockdown significantly reduced the mRNA expression of both NAT10 and KPNB1 in PC9R and A549R cells, while KPNB1 overexpression markedly increased KPNB1 mRNA levels without affecting NAT10 expression (Figure 6a and b). At the protein level, NAT10 knockdown downregulated KPNB1 and PD-L1 expression in PC9R and A549R cells, whereas KPNB1 overexpression partially restored PD-L1 protein levels without altering NAT10 expression (Figure 6c). Functionally, KPNB1 overexpression rescued the inhibition of cell proliferation induced by NAT10 knockdown in PC9R and A549R cells (Figure 6d). Additionally, the enhanced apoptosis caused by NAT10 knockdown was significantly suppressed by KPNB1 overexpression (Figure 6e). Flow cytometry analysis revealed that the increase in CD8^+^ T cells resulting from NAT10 knockdown was reversed by KPNB1 overexpression (Figure 6f). Furthermore, KPNB1 overexpression reduced the levels of TNF-α and IFN-γ, which were elevated by NAT10 knockdown (Figure 6g and h), while restoring the levels of PD-1 and TIM-3 that were suppressed by NAT10 knockdown (Figure 6i and j). Collectively, these findings demonstrate that KPNB1 overexpression restores RT resistance in NSCLC cells by reversing the enhanced immune function and cellular effects induced by NAT10 knockdown.

## 4 Discussion

Lung cancer remains the second leading cause of cancer-related mortality worldwide, with NSCLC accounting for approximately 80% of cases. Despite advances in treatment, the prognosis for NSCLC patients remains poor, characterized by a significantly low 5-year survival rate [28]. RT is a cornerstone in the treatment of various cancers, including NSCLC; however, a growing body of evidence indicates that patients often develop RT resistance over time, leading to treatment failure, metastasis, cancer recurrence, and poor prognosis. This resistance represents a major challenge in cancer therapy [29]. The development of RT resistance is closely linked to RT-induced immune dysfunction, including immunogenic cell death and depletion of T cells, which collectively maintain an immunosuppressive tumor microenvironment conducive to tumor progression [29,30]. In NSCLC, strategies targeting PD-L1 and preventing CD8^+^ T cell depletion have shown promise in overcoming RT resistance and enhancing anti-tumor immunity [17,31]. Therefore, elucidating the mechanisms underlying RT resistance, particularly those involving immune regulation, is critical for improving anti-tumor immunity and patient outcomes. In this study, we demonstrated for the first time that NAT10 expression is elevated in radiation-resistant NSCLC cells. Mechanistically, NAT10 enhances KPNB1 expression through increased ac4C modification, thereby promoting immune escape in NSCLC cells by facilitating PD-L1 nuclear translocation. This process ultimately contributes to RT resistance in NSCLC.

NAT10 is the sole known enzyme responsible for catalyzing ac4C production in eukaryotic RNA. By mediating ac4C modification, NAT10 regulates RNA stability and translation efficiency, playing a critical role in the pathogenesis of various diseases. Accumulating evidence indicates that upregulated NAT10 expression is associated with cancer development and metastasis [21–23]. Specifically, NAT10 is significantly overexpressed in patients with NSCLC and has been shown to promote the proliferation, migration, and metastasis of NSCLC cells [25]. Previous studies have reported that NAT10 regulates NSCLC progression through mechanisms involving epithelial–mesenchymal transition, cell cycle modulation, and immune cell infiltration [24,25,32]. NAT10 also plays a pivotal role in cancer immune regulation. For instance, Li et al. [33] demonstrated that NAT10 enhances YWHAH expression through ac4C modification, leading to CD8^+^ T cell depletion in colorectal cancer. Additionally, NAT10 promotes PD-L1 transcription in various cancers by facilitating NPM1 acetylation, which correlates with poor patient prognosis [34]. Notably, radiation has been shown to enhance NAT10-mediated acetylation of MORC2 in breast cancer, suggesting that NAT10 may serve as a potential therapeutic target to overcome RT resistance [35]. However, the mechanisms by which NAT10 mediates RT resistance in NSCLC remain poorly understood. In this study, we demonstrated that NAT10 is upregulated in radiation-resistant NSCLC cells, where it enhances ac4C modification and mRNA stability of KPNB1. This process promotes PD-L1 nuclear translocation and immune escape in radiation-resistant NSCLC cells. These findings establish NAT10 as a key driver of RT resistance in NSCLC.

KPNB1, a nuclear transport receptor, plays a critical role in mediating nuclear import processes [36]. It facilitates the nuclear translocation of numerous key tumor-related mediators, thereby driving tumor progression [37]. For instance, Li et al. [38] demonstrated that KPNB1 regulates the downstream expression of NLGN3 by mediating the nuclear import of YBX1, promoting the growth of glioma cells. Similarly, Du et al. [39] showed that KPNB1 facilitates the nuclear translocation of PD-L1 in NSCLC cells, enhancing their proliferation. Additionally, KPNB1 has been implicated in RT resistance. Hazawa et al. [40] reported that inhibiting KPNB1 enhances radiation-induced apoptosis and radiosensitivity while reducing PD-L1 expression on the cell surface in human head and neck squamous cell carcinoma cells.

In this study, we provide the first evidence of KPNB1’s role in RT resistance in NSCLC. We demonstrated that KPNB1 interacts with PD-L1 in radiation-resistant NSCLC cells and promotes PD-L1 nuclear translocation, thereby contributing to radiation resistance in NSCLC.

In summary, this study demonstrates that NAT10 acts as a key promoter of RT resistance in NSCLC. NAT10 enhances KPNB1 expression through ac4C modification, thereby facilitating PD-L1 nuclear translocation and promoting immune escape in radiation-resistant NSCLC cells. These findings reveal a novel mechanism underlying RT resistance in NSCLC and suggest that targeting NAT10 or its downstream pathways may provide a promising therapeutic strategy to overcome RT resistance in clinical settings.

## Supplementary Material

Supplementary Figure
