# Supplementary material for: NAT10 promotes radiotherapy resistance in non-small cell lung cancer by regulating KPNB1-mediated PD-L1 nuclear translocation
Source: Open Life Sci. 2025 Mar 18;20(1):20251065. doi: 10.1515/biol-2025-1065 (PMC11920766; doi:10.1515/biol-2025-1065)
Supplement: Supplementary Figure [file biol-2025-1065-sm.pdf]

Supplementary material

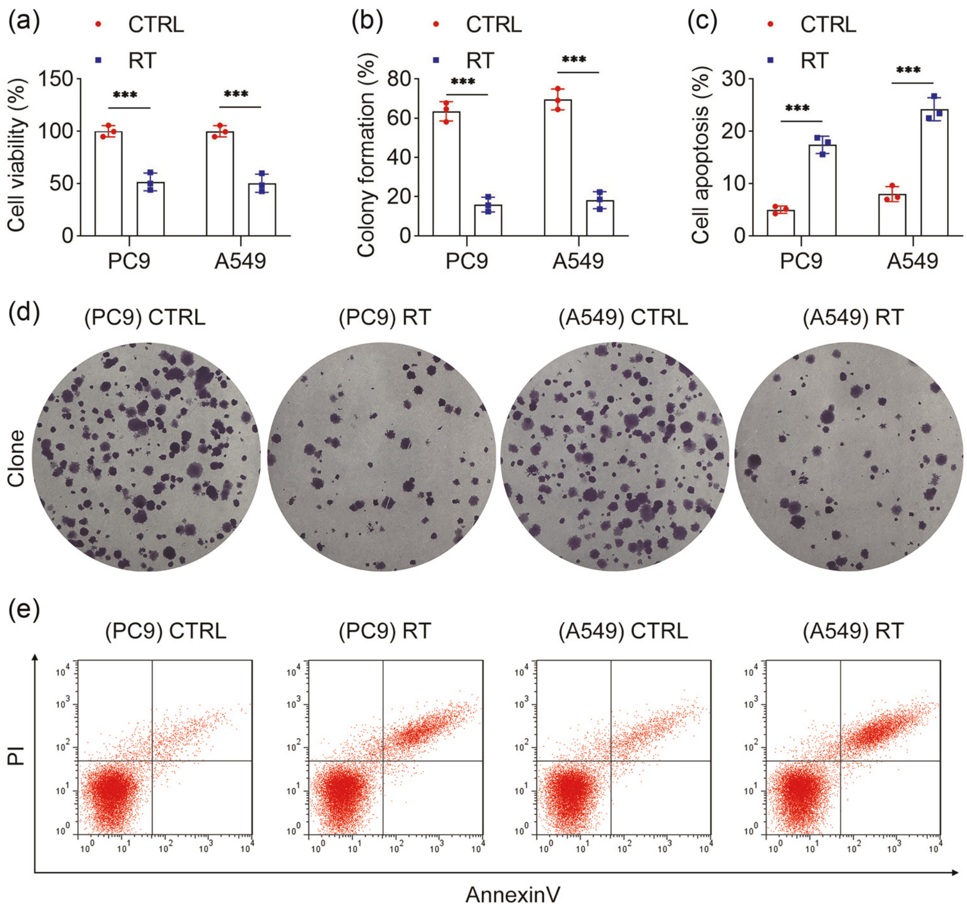

Figure S1: Effects of RT on cell viability, proliferation and apoptosis of parental PC9 and A549 cells. (a) Cell viability of PC9 and A549 cells was detected using a CCK8 kit. (b) and (d) Cell proliferation ability of PC9R and A549R cells was evaluated by colony formation assay. (c) and (e) Cell apoptosis of PC9R and A549R cells was detected by flow cytometry.

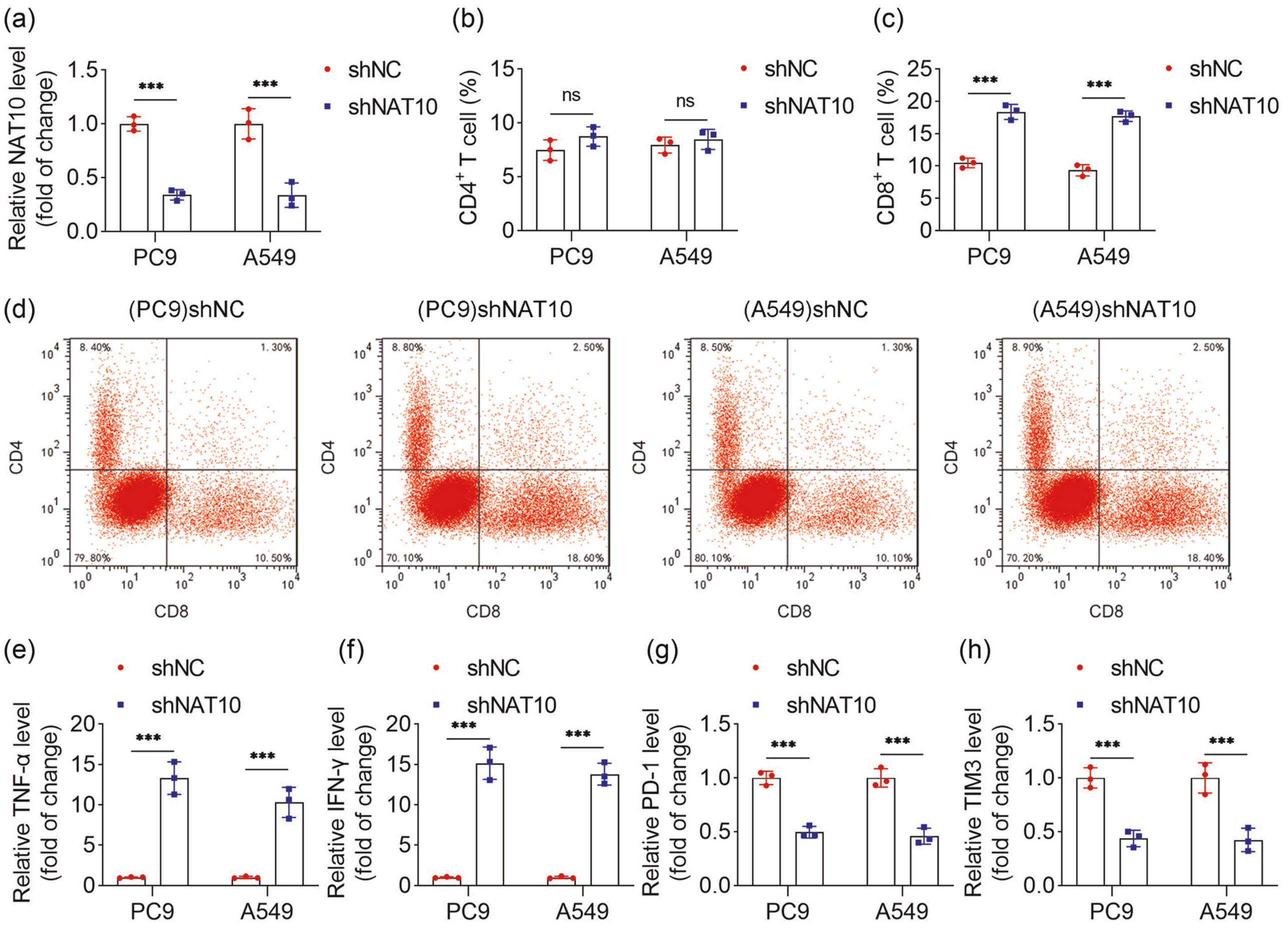

Figure S2: Effects of NAT10 knockdown on T cells proportion and levels of TNF-α, IFN-γ, PD-1 and TIM3 in co-culture of PBMCs with PC9 and A549. (a) NAT10 expression of PC9 and A549 cells was measured by qPCR. (b)–(d) Flow cytometry was performed to detect the proportion of $CD4^+$ and $CD8^+$ T cells. (e)–(h) The levels of TNF-α, IFN-γ, PD-1 and TIM3 were measured by qPCR.

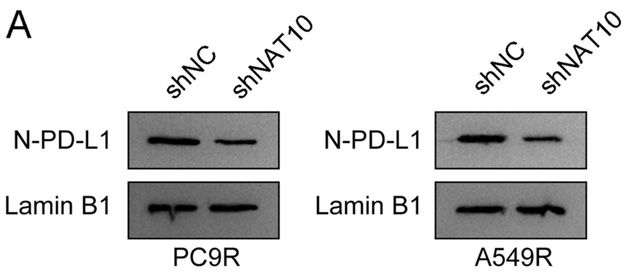

Figure S3: NAT10 knockdown downregulated the protein levels of PD-L1 in nucleus of PC9R and A549R cells. (a) Western blot was performed to detect the protein levels of PD-L1 in nucleus of PC9R and A549R cells.

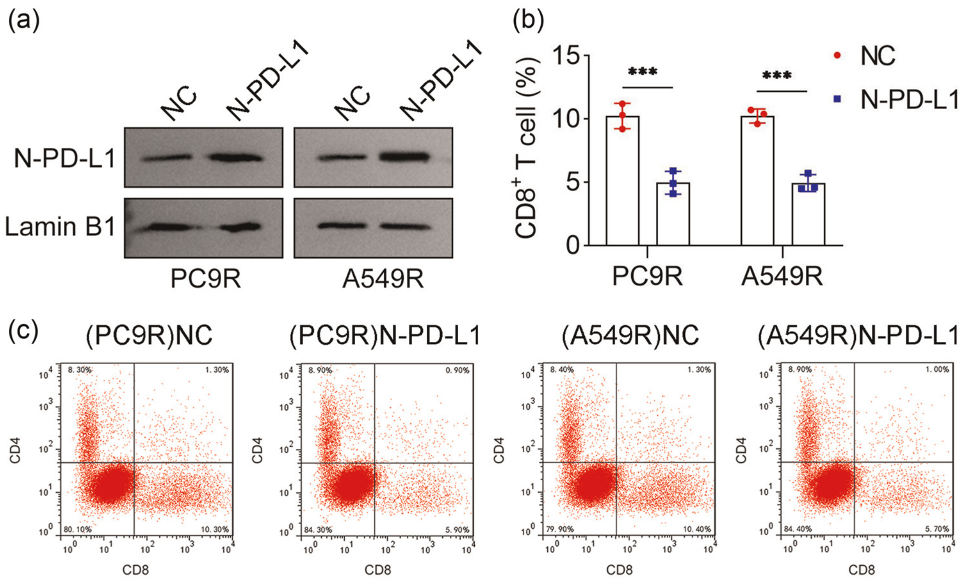

Figure S4: Overexpression of PD-L1 in nucleus of PC9R and A549R cells decreased the proportion of $CD8^{+}$ T cells. (a) The protein levels of PD-L1 in nucleus of PC9R and A549R cells were detected by western blot. (b) and (c) The proportion of $CD8^{+}$ T cells was evaluated by flow cytometry.
